# Supplementary material for: Resolving the full spectrum of human genome variation using Linked-Reads
Source: Genome Res. 2019 Apr;29(4):635–45. doi: 10.1101/gr.234443.118 (PMC6442396; doi:10.1101/gr.234443.118)
Supplement: Supplemental Material [file supp_gr.234443.118_Supplemental_longranger-2.2.zip › longranger-2.2.tar.bz2!/lr2.2-public/lib/stan/cmdstan/stan/doc/api/html/rev_2functions_2asinh_8hpp_source.html]

Stan: src/stan/agrad/rev/functions/asinh.hpp Source File


|  |  |
| --- | --- |
|  | Stan  2.5.0  probability, sampling & optimization |


- Main Page
- Related Pages
- Namespaces
- Classes
- Files

- File List
- File Members

All Classes Namespaces Files Functions Variables Typedefs Enumerations Enumerator Friends Macros Pages

- src
- stan
- agrad
- rev
- functions

asinh.hpp

Go to the documentation of this file.

1 #ifndef STAN\_\_AGRAD\_\_REV\_\_FUNCTIONS\_\_ASINH\_HPP

2 #define STAN\_\_AGRAD\_\_REV\_\_FUNCTIONS\_\_ASINH\_HPP

3

4 #include <valarray>

5 #include <stan/agrad/rev/var.hpp>

6 #include <stan/agrad/rev/internal/v\_vari.hpp>

7 #include <boost/math/special\_functions/fpclassify.hpp>

8 #include <stan/agrad/rev/numeric\_limits.hpp>

9 #include <stan/agrad/rev/operators/operator\_equal.hpp>

10 #include <stan/agrad/rev/operators/operator\_unary\_negative.hpp>

11 #include <math.h>

12

13 namespace stan {

14  namespace agrad {

15

16  namespace {

17  class asinh\_vari : public op\_v\_vari {

18  public:

19  asinh\_vari(double val, vari\* avi) :

20  op\_v\_vari(val,avi) {

21  }

22  void chain() {

23  avi\_->adj\_ += adj\_ / std::sqrt(avi\_->val\_ \* avi\_->val\_ + 1.0);

24  }

25  };

26  }

27

65  inline var asinh(const stan::agrad::var& a) {

66  if (boost::math::isinf(a.val()))

67  return var(new asinh\_vari(a.val(), a.vi\_));

68  return var(new asinh\_vari(::asinh(a.val()),a.vi\_));

69  }

70

71  }

72 }

73 #endif

stan::agrad::var::val

double val() const

Return the value of this variable.

**Definition:** var.hpp:209

operator\_equal.hpp

stan::agrad::var::vi\_

vari \* vi\_

Pointer to the implementation of this variable.

**Definition:** var.hpp:40

boost::math::isinf

bool isinf(const stan::agrad::var &v)

Checks if the given number is infinite.

**Definition:** boost\_fpclassify.hpp:54

v\_vari.hpp

stan::agrad::sqrt

fvar< T > sqrt(const fvar< T > &x)

**Definition:** sqrt.hpp:15

var.hpp

stan::agrad::asinh

fvar< T > asinh(const fvar< T > &x)

**Definition:** asinh.hpp:16

stan::agrad::var

Independent (input) and dependent (output) variables for gradients.

**Definition:** var.hpp:27

operator\_unary\_negative.hpp

numeric\_limits.hpp


---

[ Stan Home Page ]

*© 2011–2014,
Stan Development Team.*
